# Unlocking the Promise of Decellularized Pancreatic Tissue: A Novel Approach to Support Angiogenesis in Engineered Tissue

**DOI:** 10.3390/bioengineering11020183

**Published:** 2024-02-14

**Authors:** Lei Hao, Fariba Khajouei, Jaselin Rodriguez, Soojin Kim, Eun Jung A. Lee

**Affiliations:** Department of Biomedical Engineering, New Jersey Institute of Technology, Newark, NJ 07102, USA; lh34@njit.edu (L.H.); fk84@njit.edu (F.K.); jmr99@njit.edu (J.R.); sk2289@njit.edu (S.K.)

**Keywords:** tissue engineering and regenerative medicine, pancreatic ECM, decellularization, angiogenesis, biological scaffold, biomaterials

## Abstract

Advancements in regenerative medicine have highlighted the potential of decellularized extracellular matrix (ECM) as a scaffold for organ bioengineering. Although the potential of ECM in major organ systems is well-recognized, studies focusing on the angiogenic effects of pancreatic ECM are limited. This study investigates the capabilities of pancreatic ECM, particularly its role in promoting angiogenesis. Using a Triton-X-100 solution, porcine pancreas was successfully decellularized, resulting in a significant reduction in DNA content (97.1% removal) while preserving key pancreatic ECM components. A three-dimensional ECM hydrogel was then created from this decellularized tissue and used for cell culture. Biocompatibility tests demonstrated enhanced adhesion and proliferation of mouse embryonic stem cell-derived endothelial cells (mES-ECs) and human umbilical vein endothelial cells (HUVECs) in this hydrogel compared to conventional scaffolds. The angiogenic potential was evaluated through tube formation assays, wherein the cells showed superior tube formation capabilities in ECM hydrogel compared to rat tail collagen. The RT-PCR analysis further confirmed the upregulation of pro-angiogenic genes in HUVECs cultured within the ECM hydrogel. Specifically, HUVECs cultured in the ECM hydrogel exhibited a significant upregulation in the expression of MMP2, VEGF and PAR-1, compared to those cultured in collagen hydrogel or in a monolayer condition. The identification of ECM proteins, specifically PRSS2 and Decorin, further supports the efficacy of pancreatic ECM hydrogel as an angiogenic scaffold. These findings highlight the therapeutic promise of pancreatic ECM hydrogel as a candidate for vascularized tissue engineering application.

## 1. Introduction

Recent advances in regenerative medicine gave rise to a widespread interest in utilizing decellularized extracellular matrix (ECM) as a biological scaffold to develop novel treatment options [1,2,3,4,5] and suggest the possibility of employing ECM as a scaffold to bioengineer functional organs [4,6,7]. Commonly explored decellularization methods include physical [8,9], biological [10,11] and chemical [12,13,14] techniques. Physical methods such as electroporation and hydrostatic pressure are precise and non-toxic, yet their efficacy varies across tissues and may damage the ECM structure [15]. Biological approaches involve the use of enzymes like collagenase to selectively target cellular components, but they may not completely remove the cells and could damage delicate ECM structures [12]. Chemical methods utilize chemical detergents such as Sodium dodecyl sulfate (SDS) and Triton X-100. SDS is highly effective in cell removal due to its ability to solubilize cell membranes and denature proteins. However, its aggressive nature can lead to the degradation of essential ECM components [16]. In contrast, Triton X-100 is considered for its gentleness and better preservation of bioactive integrity [17]. While the efficacy for cell removal may be lower compared to that of harsher detergents, Triton X-100 is considered a preferred method for the better preservation of ECM components and functionality [14,18,19]. Thus far, successful decellularization methods and revascularization efforts of the ECM have been published in major organ systems: heart [20,21], lungs [22,23], kidney [24], and liver [25]. However, there has been limited research investigating pancreatic ECM, specifically its underlying angiogenic effect and benefits.

Researching clinical and regenerative applications of pancreatic tissue and ECM are of clinical importance; pancreatic dysregulation and dysfunction are implicated as one of the most prevalent medical conditions: diabetes mellitus (DM), a disease that is increasing in prevalence worldwide and is linked to high-risk complications, i.e., myocardial infarction, stroke, kidney disease [26]. Diabetes management is most commonly performed with insulin supplementation therapy. While a stable source of insulin through transplantation of the pancreas (and now specifically islets) provides a means to better regulate hypoglycemic levels for Type I DM patients, donor pancreatic tissue is extremely limited. A significant obstacle with islet transplantation therapies is a low survival rate/poor function of transplanted cells due to (i) ischemia and necrosis consequent to a lack of functional vasculature and (ii) pro-inflammatory immune responses against allogeneic/necrotizing transplants [27,28,29]. To address these limitations, there has been growing interest in using native pancreatic ECM as a scaffold to bioengineer pancreatic tissue or whole organs. This can be integrated with beta-cells differentiated from induced pluripotent stem cells (iPSCs) to create constructs suitable for autologous transplants [30,31,32,33]. Moreover, a recent study utilizing decellularized porcine ECM-derived microencapsulation demonstrated a significant improvement in islet function [34]. Similarly, another study incorporated human pancreatic decellularized ECM within alginate microcapsules for long-term viability and functionality of islets [35]. These advancements further support the growing importance of the pancreatic ECM not only in enhancing the islet viability and functionality but also in pancreatic tissue regeneration [36]. Considering that the pancreas is characterized by a dense and robust vascular network, and the ECM is an integral component involved in various biological processes, including the development and functioning of the pancreas [37], it is hypothesized that specific components present in the native ECM may play crucial roles in regulating angiogenesis and facilitating vascularization necessary for islet transplantation.

Thus, the overall objective of this study was to further appreciate and understand the capabilities of the pancreatic ECM, especially as a naturally derived biomaterial promoting endothelial cell growth and angiogenesis. Porcine pancreas was efficiently decellularized using an optimized decellularization protocol and characterized for its biochemical and mechanical properties. To determine the biocompatibility and cell function, decellularized porcine pancreatic ECM was reconstituted into a three-dimensional (3D) pancreatic ECM hydrogel. Mouse embryonic stem cell-derived endothelial cells (mES-ECs) and human umbilical cord vein endothelial cells (HUVECs) were then seeded into the ECM hydrogels for the evaluation of cell function. This current study demonstrates the potential of pancreas-derived ECM for facilitating vascularization in tissues. Further identification of underlying angiogenic cues that prompt vascularization will have a profound impact on the development of therapeutic applications beyond pancreatic tissues.

## 2. Materials and Methods

### 2.1. Decellularization of Pancreas

The porcine pancreas was obtained from four six-month-old pigs (Midwest Research Swine LLC, Gibbon, MN, USA). The pancreas was first washed to remove the lipid layer and the blood and cut into small pieces. Then, the material was immersed in phosphate buffer saline (PBS) under rotation for 1 h. The tissue samples were decellularized in a solution of 1% (*v*/*v*) Triton-X-100 (Boston BioProducts, Ashland, MA, USA) at 4 °C under rotation for a total of 8 h until the tissues turned translucent. The solution was changed every 30 min for the first 2 h and every 2 h after until the process was completed. The resulting ECM was washed with a large volume of sterile MilliQ (Millipore Sigma, Burlington, MA, USA) water and placed in a tube with sterile PBS to be washed for a total of 2 days with the washing solution being changed frequently for complete removal of any detergent residues remaining. The decellularized ECM was then sterilized in 70% ethanol for 2 h followed by sterile PBS wash and freeze-dried using a lyophilizer (Labconco, Kansas City, MO, USA). The sterile lyophilized tissue was kept frozen until further use.

### 2.2. Quantification of Residual DNA of Decellularized ECM

The residual DNA was quantified using a Quant-iT PicoGreen dsDNA Assay Kit (Fisher Scientific, Springfield Township, NJ, USA). Briefly, the lyophilized decellularized pancreas was dissolved in a papain digestion solution (125 mg/mL papain (Millipore Sigma, Burlington, MA, USA), 100 mM phosphate buffer, 10 mM L-cysteine Hydrochloride (Millipore Sigma, Burlington, MA, USA), and 10 mM EDTA (Millipore Sigma, Burlington, MA, USA)) at a tissue concentration of 1 mg/mL and incubated at 60 °C for 16–24 h. The absorbance of papain digest containing picogreen dye and tissue digest was measured using a microplate reader (Tecan Infinite M200, Männedorf, Switzerland). The excitation and emission wavelengths of 485 nm and 530 nm, respectively, were used. Lyophilized native pancreatic tissue was used as a control.

### 2.3. Quantification of Residual Sulfated Glycosaminoglycan (sGAG) and Collagen

The sGAG content was quantified using a dimethylmethylene blue (DMMB) assay [38]. Decellularized pancreatic tissue was lyophilized and digested in a 1 mL papain digestion solution as described above. DMMB reagent prepared with DMMB (Millipore Sigma, Burlington, MA, USA), glycine (Boston BioProducts, Ashland, MA, USA), NaCl (Millipore Sigma, Burlington, MA, USA), and acetic acid (Millipore Sigma, Burlington, MA, USA) was mixed with tissue samples in a 10:1 ratio. The absorbance was read at 525 nm using a microplate reader (Tecan Infinite M200, Männedorf, Switzerland), and the GAG content was quantified by using a chondroitin 4 sulfate (Millipore Sigma, Burlington, MA, USA) standard curve (*n* = 4). Lyophilized native pancreatic tissue was used as a control.

A hydroxyproline assay was performed to quantify the residual collagen in the porcine ECM as described previously with slight modifications [39]. All standard and tissue samples were hydrolyzed in 4 N sodium hydroxide (NaOH) and incubated at 120 °C for 3 h. Once the samples returned to room temperature, 4 N HCl was added to each sample to neutralize the pH, followed by Chloramine-T solution (0.05 M Chloramine-T in pH 6 buffer and isopropanol). Ehrlich’s solution (1 M DMAB in 30% *v*/*v* HCl and 70% *v*/*v* Isopropanol) was then added and vortexed until it formed a complete mixture and incubated at 60 °C for 90 min. The absorbance was measured at 560 nm (Biocolor Ltd., Carrickfergus, UK).

### 2.4. Mouse Embryonic Stem Cell-derived Endothelial Cells (mES-EC) Culture

The endothelial cell (EC) was derived from GFP-tagged mES cells, as previously described [40,41]. The mES-ECs were cultured in MCDB131 media (Biocompare, St. Louis, MO, USA) supplemented with 10% FBS, 1% penicillin/streptomycin, and EndoGro (VEC Technologies, Inc. Rensselaer, NY, USA) in 0.1% gelatin-coated flasks (Millipore Sigma, Burlington, MA, USA), and fresh cell culture medium was changed and replenished every 2–3 days. All cells were used at passages 7 for proliferation assay, passages 8–10 for imaging, and passages 7–10 for gene analysis.

### 2.5. Human Umbilical Vein Endothelial Cells (HUVEC) Culture

HUVECs (Lonza, Walkersville, MD, USA) were cultured in EGM™-2 BulletKit™ Medium (Lonza, Walkersville, MD, USA). Cells were sub-cultured at 70–85% confluency using 0.05% Trypsin-EDTA (Gibco, Grand Island, NY, USA) as previously described [42]. Cells under passage 4 were used for the study.

### 2.6. Preparation of 3D Decellularized Tissue Derived ECM Hydrogel

The ECM hydrogel was prepared following a protocol described by Freytes et al. [43]. Briefly, 3.57 mg of lyophilized pancreatic ECM powder was digested in pepsin/HCl in a 10:1 ratio *w*/*v* in 0.01 N HCl at room temperature for 24 h on Benchmixer vortex mixer (Benchmark Scientific, Edison, NJ, USA). Once fully digested, a viscous pre-gel solution was formed. The pre-gel solution was then mixed with 10× reconstitution buffer (0.05 N NaOH (Millipore Sigma, Burlington, MA, USA) with 0.16 M HEPES (Millipore Sigma, Burlington, MA, USA), 0.25 M NaHCO3, 5× DMEM (Gibco, Grand Island, NY, USA)), and culture media in a 70:10:10:10 ratio, respectively. Three-dimensional Type I collagen gels were prepared to use as a 3D control. Rat-tail collagen type I (BD Biosciences, Bedford, MA, USA) solution was prepared with collagen, DMEM, reconstitution buffer, and culture media in a ratio of 65:20:10:5. The final concentration of the ECM and collagen gels was 2.5 mg/mL. A cell density of 1 × 10^6^ cells/mL was used in both pancreatic ECM and collagen hydrogels. Samples were incubated at 37 °C for 2 h for complete polymerization. Culture media was then carefully added, and the hydrogels were incubated until further characterization for up to 7 days. Culture media was changed every other day.

### 2.7. Cell Proliferation Assay

To compare and quantify the proliferation of cells, hydrogels were formed with and without the cells and monitored for 5 days. A two-dimensional cell culture condition was used as a control. Cell proliferation was quantified with a Cell Proliferation Kit II (XTT) (Roche, Indianapolis, IN, USA) as described previously [44]. To perform the assay, 50 µL of XTT labeling component was directly added to the culture media of each well-containing gel or cell. Samples were incubated for 3 h at 37 °C in dark. Supernatants from each sample were collected and added to a 96-well plate for analysis. The absorbance was measured using an automatic microplate reader (Tecan Infinite M200, Männedorf, Switzerland) at timepoint 0 and, subsequently, every 24 h for up to 5 days. Net absorbance was obtained by subtracting the absorbance at 620 nm from that at 450 nm. The mean cell proliferation at each timepoint was expressed as a fold increase of net absorbance compared to that of the net absorbance at timepoint 0.

### 2.8. RT-PCR

Total RNA was extracted and purified from pancreatic ECM hydrogels seeded with HUVECs using the GenElute Mammalian Total RNA Miniprep Kit following the manufacturer’s instructions (Fisher Scientific, Springfield Township, NJ, USA). cDNA was created with 500 ng of RNA and the High-Capacity cDNA Reverse Transcription Kit (Applied Biosystems, Waltham, MA, USA) in the T100 Thermal Cycler (Bio-rad, Hercules, CA, USA). The primers used in the study are listed in Table 1 (Integrated DNA Technologies, San Diego, CA, USA) [45,46]. The qPCR reactions were prepared using SSo Advanced SYBRgreen Mastermix (Bio-rad, Hercules, CA, USA) ran in a CFX Connect Real Time System (Bio-rad, Hercules, CA, USA) for up to 40 cycles. Relative gene expression was normalized to the housekeeping gene GAPDH and presented as previously described [47].

### 2.9. Tube Formation Assay

HUVECs (3 × 10^4^ cells/cm^2^) were seeded onto Matrigel-coated wells and incubated at 37 °C. Brightfield images were captured 24 h after seeding using a Cytation 1 Cell Imaging Multi-Mode Reader (BioTek, Winooski, VT, USA). The total tube length was analyzed by utilizing the Angiogenesis Analyzer tool in Image J 1.52K [48].

### 2.10. Immunofluorescence (IF) Microscopy

Samples were fixed on day 14 with 4% paraformaldehyde for 4–6 h at room temperature. Samples were stained with Rhod19amine-phalloidin (Life Technologies, Eugene, OR, USA) and DAPI (Vector Laboratories, Burlingame, CA, USA) to visualize the F-actin and the nuclei, respectively. The fluorescent images were captured using a disk-spinning fluorescent microscope (Olympus, Somerset, NJ, USA). A LIVE/DEAD Viability/Cytotoxicity kit (Thermo Fisher, Waltham, MA, USA) was used to evaluate the viability of cells in the ECM hydrogel following the manufacturer’s instructions. To visualize PE-CAM, samples were blocked in goat serum (10% *w*/*v*), incubated with primary mouse monoclonal anti-rat PE-CAM (Dilution 1:400, Santa Cruz Biotechnology, Dallas, TX, USA) overnight at 4 °C, and incubated with secondary Alexa Fluor 647 goat anti-rat IgG (dilution 1:100, Santa Cruz Biotechnology) each for 2 h at room temperature.

### 2.11. Scanning Electron Microscopy (SEM) Analysis

To obtain SEM images, cell-seeded pancreatic ECM hydrogels were first fixed in a 2% (*v*/*v*) glutaraldehyde solution (Millipore Sigma-Aldrich, Burlington, MA, USA) at day 14 of the culture. Samples were then dehydrated using ethanol, dried using the critical point drying (CPD) method with a Tousimis Samdri 790 machine (Tousimis Research, Rockville, MD, USA) and sputter coated with 6 nm Au-Pd with an EMS 150T ES machine (Quorum Technologies, Sacramento, CA, USA). SEM images were acquired using a Schottky Field Emission Scanning Electron Microscope (Joel JSM 7900F Peabody, MA, USA).

### 2.12. Rheological Analysis

To assess the biomechanical properties of the cell-seeded pancreatic ECM hydrogels, rheology experiments were performed on fully formed hydrogels after 3 days of culture. Discovery HR-2 rheometer (TA Instruments, New Castle, DE, USA) with 8 mm plate-geometry was used at room temperature. The optimal gap width between the shearing plates was determined to be 1 mm to confine the hydrogel. A strain sweep using 0.01 to 10% strain was performed at a fixed frequency of 0.1 Hz to determine the storage (G′) and loss modulus (G″).

### 2.13. Statistical Analysis

Statistical analyses were performed using Prism 10 (GraphPad Software, Inc., Boston, MA, USA). Results are presented as mean ± SD. Statistical comparisons on paired data were performed using a student *t*-test [49]. For multiple comparisons of parametric data, ANOVA [50] and Bonferroni’s post hoc test [51] were used (*p* < 0.05 considered significant).

## 3. Results

**Figure 1:**
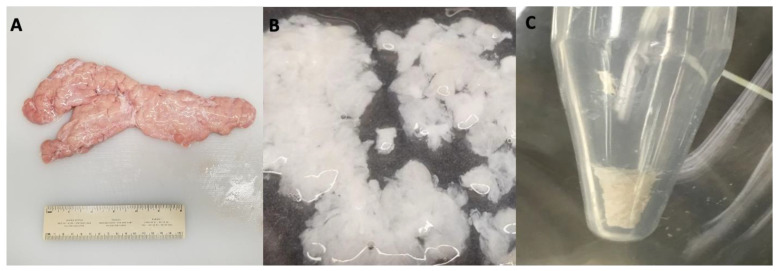
Images demonstrating the key steps of decellularization of pancreatic tissue: (**A**) a whole porcine pancreas, (**B**) sectioned pancreatic tissue post-decellularization process, and (**C**) decellularized pancreatic ECM after lyophilization.

Porcine pancreas (Figure 1A) was successfully decellularized using a Triton-X-100 solution, followed by extensive washing to remove the remaining detergent and cellular debris. Upon completion of decellularization, the resulting tissue became translucent, as shown in Figure 1B. Figure 1C demonstrates lyophilized decellularized pancreatic tissue.

**Figure 2:**
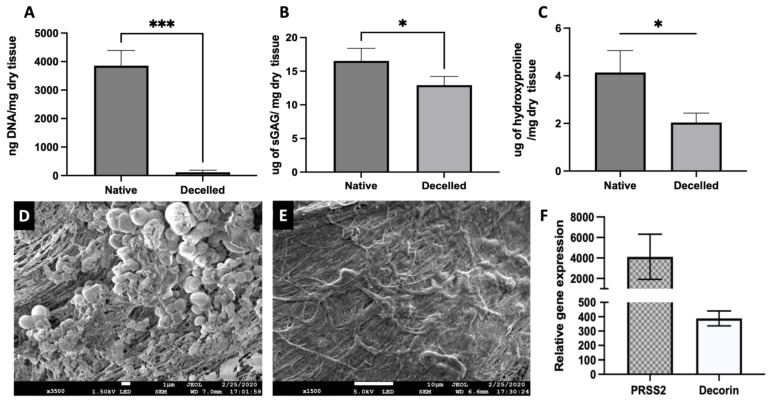
Biochemical and morphological characterization of native and decellularized pancreatic tissue. Figures demonstrate the quantitative analysis of (**A**) double-stranded DNA, (**B**) Sulfated Glycosaminoglycan (sGAG), and (**C**) Hydroxyproline in the native tissue compared to the decellularized tissue. (paired *t*-test, *n* = 4, mean ± SD, * *p* < 0.05, *** *p* < 0.001). Scanning electron microscopy images of native vs. decellularized porcine pancreatic tissue (**D**) and (**E**), respectively. (**F**) The RT-PCR results confirmed the expression of PRSS2 and Decorin, recognized ECM proteins abundantly found in human and porcine pancreases in decellularized pancreatic tissue (*n* = 3, mean ± SD).

DNA assay revealed a significant decrease in the amount of dsDNA in the decellularized tissue (111.1 ± 76.3 ng DNA per mg dry tissue) compared to that of the native pancreas (3856 ± 533.3 ng DNA per mg dry tissue), resulting in 97.1% removal of DNA (Figure 2A). The DMMB assay revealed a significant reduction; however, 78% of sGAG was retained following decellularization (16.5 ± 1.9 µg per mg dry tissue vs. 12.9 ± 1.3 µg) (Figure 2B). Hydroxyproline assay also demonstrated that approximately 50% of the collagen content was preserved post-decellularization in comparison to the native tissue (Figure 2C). Further, unlike the native tissue exhibiting high cell presence (Figure 2D), no cells were visible on the surface of decellularized ECM (Figure 2E), as demonstrated in the SEM images. To further evaluate the preservation of proteinaceous content in decellularized pancreatic ECM, PRSS2 and Decorin, which are the known ECM proteins abundantly present in both human and porcine pancreases, were examined. The RT-PCR results demonstrated that the decellularized porcine pancreas expresses both ECM proteins, as shown in Figure 2F (*n* = 3).

**Figure 3:**
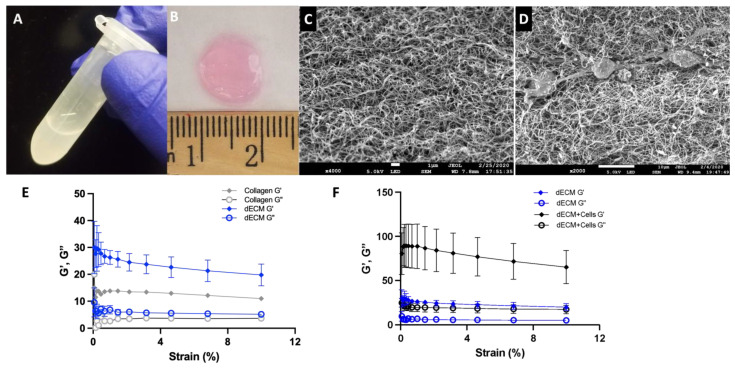
Macroscopic images showing solubilized pancreatic ECM in pepsin-HCl (**A**) and a 3D reconstituted ECM hydrogel (**B**). SEM images illustrating the structure of pancreatic ECM hydrogel without cells (**C**) and with mES-ECs (**D**). (**E**) Comparison of the mechanical properties between pancreatic ECM hydrogels and Type I collagen, and (**F**) the impact of cells on pancreatic ECM hydrogels (G′: elastic modulus, G″: viscous modulus).

After successful decellularization and lyophilization, the pancreatic tissues were digested in pepsin/HCL, as shown in Figure 3A. The resulting pancreatic ECM solution was then successfully reconstituted into a 3D hydrogel, as demonstrated in Figure 3B. Pancreatic ECM hydrogels exhibited uniform fibrous structures and networks, as evidenced by the SEM image (Figure 3C). Similar fibrous networks were observed in pancreatic hydrogels seeded with endothelial cells (Figure 3D), confirming that the porous and fibrous networks are well preserved in hydrogels formed from decellularized tissue. To evaluate the mechanical properties of the pancreatic hydrogels, a strain sweep test was performed at 0.1 Hz frequency using a rheometer. The rheology experiments revealed that the storage modulus of pancreatic ECM hydrogels and rat tail collagen hydrogels is comparable, as shown in Figure 3E. Upon the addition of cells to the pancreatic ECM hydrogel, the G′ value significantly increased compared to ECM hydrogels without cells (unloaded, 65.25 ± 18.8 Pa vs. 19.36 ± 3.62 Pa), indicating that cells contribute to the stiffening of the pancreatic ECM hydrogel (Figure 3F).

**Figure 4:**
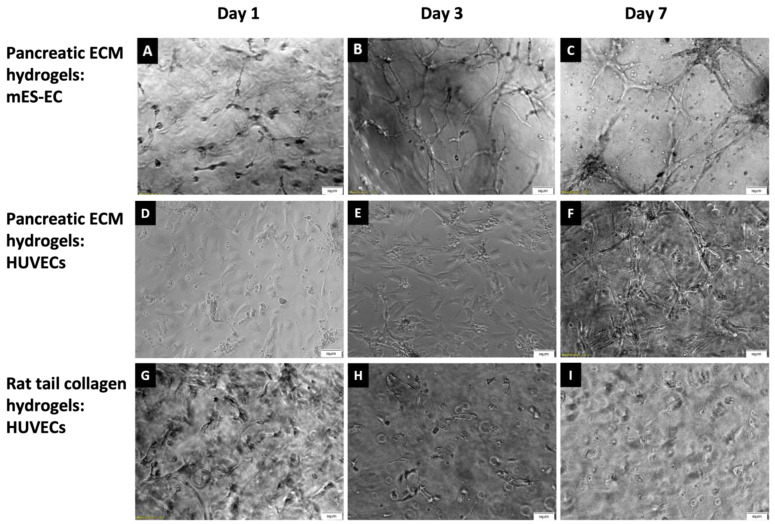
Phase-contrast images demonstrating mES-ECs (1 × 10^6^ cells/mL) in pancreatic ECM at days 1 (**A**), 3 (**B**) and 7(**C**). HUVECs (1 × 10^6^ cells/mL) seeded in pancreatic ECM are presented on days 1 (**D**), 3 (**E**) and 7 (**F**). Additionally, HUVECs in Type I collagen are presented at days 1 (**G**), 3 (**H**) and 7 (**I**), respectively. Scale bar = 50 μm.

The biocompatibility of pancreatic ECM hydrogels was further determined using two distinct endothelial cell types: mES-ECs and HUVECs. Both cell types adhered and spread within the pancreatic ECM hydrogels, as shown in Figure 4. Cells in Type I collagen gel, however, exhibited less cell spreading over the 7-day culture period compared to pancreatic ECM hydrogel. Moreover, the formation of capillary-like structures was evident in both cell types only within the pancreatic ECM hydrogel, not in Type I collagen hydrogels, after 7 days of culture (Figure 4C,F,I).

**Figure 5:**
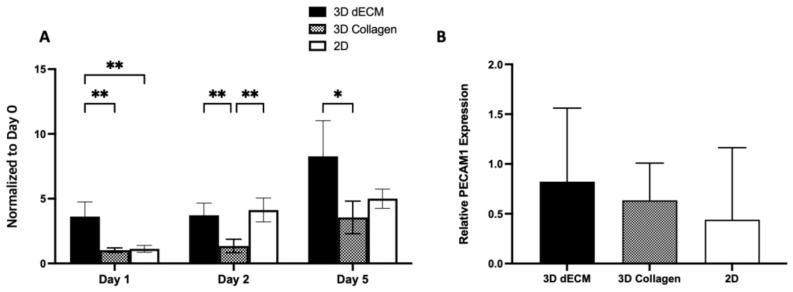
(**A**) Quantitative analysis of mES-ECs proliferation in pancreatic ECM, collagen hydrogels and 2D culture at day 1, day 2, and day 5 normalized to day 0. (**B**) The expression of PE-CAM1 by mES-ECs in pancreatic ECM hydrogel, collagen hydrogels and two-dimensional culture (*n* = 3, mean ± SD, * *p* < 0.05, ** *p* < 0.01).

Endothelial cell proliferation was also evaluated using the XTT assay over a 5-day culture period. The results revealed that mES-ECs exhibited significantly higher proliferation in pancreatic ECM hydrogel compared to cells cultured in Type I collagen hydrogels or on tissue culture plates at day 1 (Figure 5A). Proliferation continued in all conditions, with notable cell proliferation observed in pancreatic ECM hydrogel compared to Type I collagen hydrogels at day 5. Western blot results confirmed the expression of PE-CAM1 by mES-ECs in all experimental conditions, including pancreatic ECM hydrogel culture. This suggests that the endothelial cell phenotype is well maintained in the 3D pancreatic ECM hydrogel (Figure 5B).

**Figure 6:**
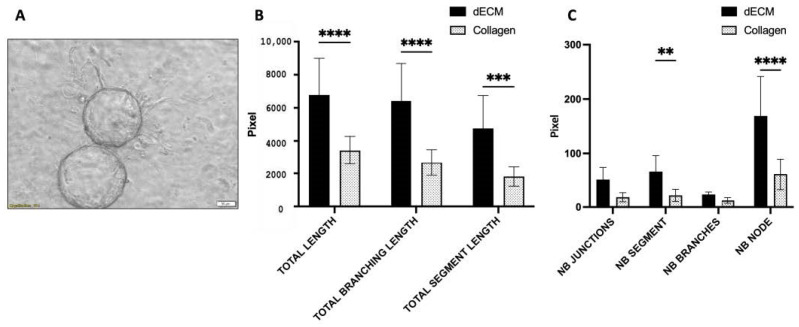
(**A**) Phase-contrast image of HUVECs on microbeads within the decellularized pancreatic ECM (dECM) hydrogel, sprouting after 24 h of culture for the angiogenesis assay (scale bar = 50 μm). (**B**) Quantitative analysis revealed that cells cultured in the pancreatic ECM hydrogel exhibit significantly greater total lengths, total branching lengths, and total segment lengths of the tubules. (**C**) Additionally, the number of segments and nodes in the pancreatic ECM is significantly higher compared to those cultured in Type I collagen gel (*n* = 6, mean ± SD), ** *p* < 0.01, *** *p* < 0.001, **** *p* < 0.0001).

Tube formation assays were performed using HUVECs to evaluate the angiogenic potential of pancreatic ECM. This assay captures the propensity of HUVEC-coated beads to form capillary-like tubules, as shown in Figure 6A. The effects of the pancreatic ECM hydrogel on the HUVECs were characterized by measuring the extent of tube formation, including the total length of tubules formed, number of segments, number of branches, number of junctions, number of nodes, and number of segments. HUVECs seeded in rat tail Type I collagen gel served as the control. The analytical assessment presented in Figure 6B,C clearly demonstrated that cells cultured in the pancreatic ECM hydrogel exhibit a markedly enhanced ability to form tubes, as evidenced by significantly greater total lengths, total branching lengths, and total segment lengths of the tubules compared to those cultured in Type I collagen gel (Figure 6B). The number of branches and nodes in the pancreatic ECM was also significantly higher, indicating a more robust network of vascular-like structures (Figure 6C).

Extended from the tube formation assay, the function of endothelial cells in the pancreatic ECM hydrogel was evaluated using RT-PCR. Samples of HUVECs in pancreatic ECM hydrogel and Type I collagen were collected after 3 days of culture, with HUVECs cultivated in a monolayer serving as a control. Results revealed that HUVECs in a monolayer express a basal level of VEGF, consistent with previous findings demonstrating VEGF expression by HUVECs in normoxic conditions [52].

**Figure 7:**
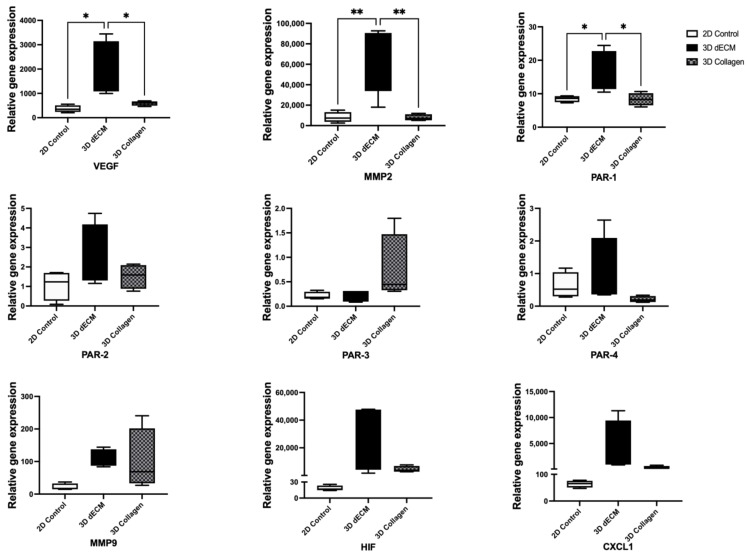
Gene expression profiles of HUVECs cultured in 2D (control), 3D decellularized pancreatic ECM hydrogels and 3D Type I collagen for 3 days. HUVECs cultured in 3D ECM exhibited a significant upregulation in the expression of MMP2, VEGF and PAR-1, compared to those cultured in collagen hydrogel or in a two-dimensional condition (*n* = 4, one-way ANOVA, * *p* < 0.05, ** *p* < 0.01).

In the ECM hydrogels, HUVECs exhibited a significant upregulation in the expression of MMP2, a crucial enzyme for matrix proteolysis and angiogenesis promotion, along with other pro-angiogenic related genes such as VEGF and PAR-1, compared to cells cultured in other conditions [53,54] (Figure 7). The expression levels of other protease-activated receptors (PAR-2, PAR-3 and PAR-4), extracellular matrix remodeling-related gene MMP9, hypoxia-associated regulator HIF and inflammatory response-related gene CXCL1 did not show significant differences among the groups. Taken together, these findings demonstrate that the angiogenic potential of endothelial cells is significantly enhanced in pancreatic ECM hydrogels compared to collagen gels, likely due to ECM hydrogel fostering an environment conducive to pro-angiogenic activity.

## 4. Discussion

Artificial pancreatic tissues require biomaterials that can properly integrate with the native pancreas, a highly vascularized and metabolically demanding organ. Thus, this study aimed to explore the potential of decellularized pancreatic ECM as a promising material for creating a proangiogenic microenvironment conducive to the growth of vascular endothelial cells. While studies have investigated the use of decellularized hydrogels reconstituted from various tissues, such as the heart, small intestinal submucosa (SIS), and diaphragmatic muscle, to investigate angiogenesis both in vitro and in vivo [55,56,57], the application of pancreatic tissue has been limited. Given the pancreas’s densely vascularized network, where islets receive up to 15% of the total blood circulation to the pancreas while constituting only 1% of the organ mass [58], it can be postulated that the pancreatic ECM offers a more favorable environment for vascular formation.

Since decellularization protocols depend on the tissue source and specific tissue engineering applications for effective decellularization of the porcine pancreas, our decellularization protocol was first optimized based on a previous study [59]. To avoid potential detrimental effects on gel formation properties, TritonX-100 was utilized instead of sodium dodecyl sulfate (SDS). The treatment duration was shortened from 24 h to 8 h, with more frequent changes of TritonX-100 solution during the decellularization process, resulting in the efficient removal of DNA, which was within the well-accepted range reported in the literature [60]. In addition, sGAG and collagen content following decellularization were well maintained. The preservation of sGAG content, especially, was comparable to that reported in previously studied tissues such as the human pancreas (19.6% and 15.2% of native tissue for non-homogenized and homogenized pancreas [61]), cornea (36% of native tissue) [62], and cartilage (20% of native tissue) [63]. Since sGAG plays a crucial role in fibrillogenesis, the mechanism by which the ECM hydrogels form [64,65], preserving sGAG concentration is critical to support the gelation of dissolved tissue into ECM hydrogel [65,66].

Beyond the assessment of sGAG and collagen, this study examined other ECM proteins in decellularized pancreatic ECM and confirmed the presence of PRSS2 and Decorin. Characterizing the ECM provides crucial insights into ECM composition, aiding in the identification of potential therapeutic targets. PRSS2, predominantly found in pancreatic juice, is typically regarded as an ECM protein highly expressed in the pancreas but not in other tissues [67]. Both PRSS2 and Decorin are closely associated with the health and function of the pancreas [67,68,69]. PRSS2 may play a role in the regulatory mechanisms of angiogenesis by interacting with and activating other proteins or growth factors, fostering an environment conducive to the growth and development of new blood vessels [67,70]. Moreover, a previous study has shown that Decorin contributes to the tube formation of HUVECs [71]. Therefore, the identification of Decorin and PRSS2 in the decellularized pancreatic ECM provides an important link between ECM proteins and angiogenesis.

Among the numerous proteins reported in pancreas tissue through proteomic analyses of human [72] and mouse pancreatic islets [73], the most abundant ECM proteins are summarized in Table 2. In addition, proteins known to play a role in angiogenesis are presented, with the indications for those that are pro-angiogenic (+) or anti-angiogenic (−) and have a dual role that stimulates/inhibits angiogenesis (±) [74]. Pancreatic islets do not deposit basement membrane proteins (BM), while ECs deposit BM proteins such as collagen, laminins, fibronectin, perlecan, and nidogens [75]. These constituents not only play an active role in islet survival and function but also emerge as regulators of angiogenesis. Our study extends this knowledge base by examining the ECM composition of the porcine pancreas, an area less explored compared to the human and mouse pancreas [76]. Porcine pancreas shares significant anatomical and functional similarities with the human organ, making it an ideal model for studying pancreatic diseases and therapeutic approaches [77].

With the decellularized pancreatic tissue, 3D ECM hydrogels were successfully created, similar to previously reported hydrogels that used different tissue sources and various decellularization methods [3,78,79]. The ECM hydrogels demonstrated proper biochemical and structural properties, making them suitable as a hydrogel scaffold. While the storage modulus is lower than the values reported for the native pancreas (0.8–1.5 kPa) [80,81], it is similar to the values reported for pancreatic ECM hydrogels (89 ± 27 Pa [3] and 21.44 ± 0.77 Pa) [59]. Moreover, the reconstituted pancreatic ECM hydrogels exhibited a porous, fibrous, and homogenous structure, consistent with other decellularized hydrogels such as the kidney [82] and dermis [79].

When endothelial cells were seeded into pancreatic ECM hydrogels, they not only spread well and proliferated but also sprouted into capillary-like structures, as demonstrated by the angiogenesis assay. Following a 3-day culture in 3D pancreatic ECM hydrogel, HUVECs exhibited a significantly upregulated expression of MMP2 and VEGF, both of which were recognized as pro-angiogenic genes [53,54], compared to the cells cultured in collagen hydrogel or in a monolayer. Furthermore, PAR-1 expression was also significantly upregulated in the HUVECs cultured in the pancreatic ECM hydrogel.

This finding is particularly intriguing when considering the dual role of the PAR-1 gene in angiogenesis. Specifically, PAR-1 not only stimulates the release of VEGF but is also linked to the heightened activity of MMP-2 [83,84]. The increased MMP-2 activity suggests accelerated ECM remodeling, which is essential for endothelial cell migration and new vessel formation [85]. Concurrently, the upregulation of VEGF, driven by PAR-1, promotes endothelial cell growth, further facilitating angiogenesis [86]. These interconnected mechanisms underscore the efficacy of pancreatic ECM hydrogel in creating an angiogenic environment, highlighting its potential in tissue engineering applications.

More interestingly, the results of gene expression analysis are connected to the ECM protein identified in our study. Specifically, PRSS2 encodes for trypsinogen, which can subsequently convert into trypsin [67]. A previous study demonstrated that mesotrypsin (a recombinant trypsin isoform) can activate PAR-1 [70]. This link between PAR-1 and PRSS2, along with the role of PAR-1 on the expression of both VEGF and MMP2, implies a possible correlation between PRSS2 and PAR-1. Thus, the observed upregulation of PAR-1 in HUVECs might be attributed to the presence of PRSS2 in the ECM hydrogel.

In summary, our study demonstrates the porcine pancreatic ECM hydrogel as a platform for supporting endothelial cell growth and vascular formation. Given the need to establish vasculature in engineered tissues as well as in islets/beta-cell transplantations, the utilization of pancreas-derived ECM can serve as a valuable tool. Future research awaits in vivo applications with the aim of optimizing the methods for eventual clinical use.

## Figures and Tables

**Table 1:** List of primers used in RT-PCR.

Gene	Primer Sequence
GAPDH fw (human)	5′-CAT GGC CTT CCG TGT TCC TA-3′
GAPDH rev (human)	5′-CCT GCT TCA CCA CCT TCT TGA T-3′
MMP-2 fw	5′-TGGCAAGTACGGCTTCTGTC-3′
MMP-2 rev	5′-TTCTTGTCGCGGTCGTAGTC-3′
MMP-9 fw	5′-TGCGCTACCACCTCGAACTT-3′
MMP-9 rev	5′-GATGCCATTGACGTCGTCCT-3′
VEGF-A fw	5′-CGGCGAAGAGAAGAGACACA-3′
VEGF-A rev	5′-GGAGGAAGGTC- AACCACTCA-3′
CXCL1 fw	5′-GCGCCCAAACCGAAGTCATA-3′
CXCL1 rev	5′-ATGGGGGATGCAGGATTGAG-3′
HIF-1A fw	5′- GAAAGCGCAAGTCTTCAAAG-3′
HIF-1A rev	5′-TGGGTAGGAGATGGAGATGC-3′
GAPDH fw (porcine)	5′-TCGGAGTGAACGGATTTG-3′
GAPDH rev (porcine)	5′-CCTGGAAGATGGTGATGG-3′
Decorin fw	5′-GATGCAGCTAGCCTGAAAGG-3′
Decorin rev	5′-TCACACCCGAATAAGAAGCC-3′
PRSS2 fw	5′-TCACCTGCGGTCCTCAATTC-3′
PRSS2 rev	5′-TATGAGGCTTCACACTCGGC-3′
PAR-1 fw	5′-TGTGAACTGATCATGTTTATG-3′
PAR-1 rev	5′-TTCGTAAGATAAGAGATATGT-3′
PAR-2 fw	5′-AACATCATGACAGGTCGTGAT-3′
PAR-2 rev	5′- AGAAGCCTTATTGGTAAGGTT-3′
PAR-3- fw	5′-CTGATACCTGCCATCTACCTCC-3
PAR-3 rev	5′- AGAAAACTGTTGCCCACACC-3′
PAR-4 fw	5′- ATTACTCGGACCCGAGCC-3
PAR-4 rev	5′-TGTAAGGCCCACCCTTCTC-3′

**Table 2:** List of most abundant ECM proteins in the pancreas/Islets.

Gene	Protein	Human Pancreas [44]	Mouse Islets [45]	Role in Angiogenesis [46]
COL1 (A and B)	Collagen I	✓	✓	+
COL3	Collagen III	✓	✓	
COL6	Collagen VI	✓	✓	
REG1A	Regenerating Family Member 1 Alpha	✓	✓	
HSPG2	Perlecan	✓	✓	±
LAMC1	Laminin Gamma 1	✓	✓	±
DCN	Decorin	✓	✓	±
LUM	Lumican	✓	✓	−
COL4	Collagen IV	✓	✓	±
FBN2	Fibulin 2	✓	✓	
COL5	Collagen V	✓	✓	
LAMA2	Laminin Alpha-2	✓	✓	±
LAMB1	Laminin Beta-1	✓	✓	±
ANXA2	Annexin A2	✓	✓	
COL14A1	Collagen XIV	✓	✓	
LAMA5	Laminin Apha-5	✓	✓	±
CTSD	Cathepsin D	✓	✓	
COL18A1	Collagen Alpha-1(XVIII) chain	✓	✓	−
BGN	Biglycan	✓	✓	+
ANXA4	Annexin A4	✓	✓	
PRSS2	Anionic Trypsinogen	✓	✓	

## Data Availability

All data created or analyzed in this study are available on request from the corresponding author.
